# The Influence of Maternal Obesity on Cell-Free Fetal DNA and Blood Pressure Regulation in Pregnancies with Hypertensive Disorders

**DOI:** 10.3390/medicina57090962

**Published:** 2021-09-12

**Authors:** Aleksandra Stupak, Wojciech Kwaśniewski, Anna Goździcka-Józefiak, Anna Kwaśniewska

**Affiliations:** 1 Department of Obstetrics and Pathology of Pregnancy, Medical University of Lublin, 20-081 Lublin, Poland; anna.kwasniewska@umlub.pl; 2 Department of Gynecology Oncology and Gynecology, Medical University of Lublin, 20-081 Lublin, Poland; wojciech.kwasniewski@umlub.pl; 3 Department of Molecular Virology, Institute of Experimental Biology, Adam Mickiewicz University in Poznań, 61-614 Poznań, Poland; anna.gozdzicka-jozefiak@amu.edu.pl

**Keywords:** obesity, preeclampsia, hypertension, placenta, apelin, prosalusin, salusin, cell-free fetal DNA

## Abstract

*Background and Objectives:* obesity and blood pressure disorders are one of the main risk factors for antenatal, intra, postpartum, and neonatal complications. In preeclampsia (PE), the placental hypoxia leads to vascular endothelium dysfunction, cell necrosis, and apoptosis. This condition is associated with the release of free fetal DNA (cffDNA) circulating in plasma. The disturbance of the efficiency of vasodilatation and blood pressure regulation in PE can be confirmed by analyzing the apelin, salusin, and prosalusin. This study aimed to assess the influence of obesity on cffDNA, and the effectiveness of maintaining normal blood pressure in patients with preeclampsia and gestational hypertension. *Material and Methods*: the research material was blood serum and oral mucosa swabs, obtained from 168 patients. Pregnant women were divided into the following: a control group (C)—67 women; a gestational hypertension group (GH)—35 patients; a preeclampsia with obesity group (PE + O) (pre-gravid BMI > 30)—23 patients. The rest were lean preeclamptic women (PE)—66 patients—(pre-gravid BMI < 25 in 43 women). *Results:* the cffDNA was observed in 1.50% of women in the C group, in 2.45% in the GH group, but in 18.18% of lean patients with preeclampsia. The cffDNA was detected in 58% of obese pregnant women with PE. The greater the placental hypoxia was in preeclampsia, the less efficient the hypotensive mechanisms, according to an analysis of the studied adipokines. The prosalusin concentration was significantly lower in the PE group with cffDNA than in the PE group without it (*p* = 0.008). Apelin was higher in the PE group with cffDNA *(p* = 0.006) compared to other groups. The same results were also observed in the subgroup with obesity. *Conclusion*: in preeclamptic women, obesity seems to act as an additive factor of placental damage by means of the dysregulation of hypotensive mechanisms.

## 1. Introduction

Obesity in pregnant women is associated with a significant risk of gestational diabetes, blood pressure disturbance, fetal growth restriction or over nutrition, birth complications and an increased number of caesarean sections. Therefore, in recent decades, it has been a particular concern in global perinatal health care [1]. A high body mass index (BMI) in pregnant women may reduce the chance of effective screening (traditional serum analysis and ultrasound). For this reason, the multifactorial prognostic tests are the most effective option for the anticipation of particular pregnancy complications.

The influence of obesity on the course of pregnancy is mediated by the action of adipocytes. The cells secrete hormones, which regulate metabolism, and may increase the production of pro-inflammatory agents, regulate reproductive functions, vasoconstriction, and dilatation. Thus, fat tissue has a major role in processes involved in the pathogenesis of preeclampsia.

Hypertensive disorders of pregnancy are classified into one of several categories: chronic hypertension present before 20 weeks gestation, preeclampsia–eclampsia, chronic hypertension with superimposed preeclampsia and gestational hypertension. The hypothesis of inadequate trophoblast invasion leading to incomplete remodeling of the uterine spiral arteries has been considered to be a primary cause of the placental ischemia/hypoxia. However, at present, other interesting cardiovascular effector systems are being researched, to see whether they are responsible for smooth muscle relaxation in the systemic vessels. 

The efficiency of the hypotensive mechanisms, such as vasodilatation and blood pressure regulation, in the pregnant body can be confirmed by adipokines such as apelin and salusin. Apelin, identified in 1998, is a peptide encoded by the APLL gene and an endogenous ligand for angiotensin receptor-like 1 (APJ receptor) [2]. This protein is found on the surface of some of the body’s cells: the endothelium, heart, kidneys, liver, and adrenal glands [3]. The expression of this receptor in the blood vessels is involved in the control of the blood pressure and angiogenesis. Apelin’s antihypertensive effect consists of activating the endothelial receptors and inducing the release of nitric oxide [4]. The proangiogenic function can be summarized as increasing the proliferation and migration of endothelial cells. By participating in the migration of progenitor cells, which then form adipocytes, apelin affects the inotropic function of the positive myocardium as a mediator in the control of blood pressure and cardiovascular flow. In addition, the peptide regulates the body’s fluid management and glucose metabolism. Apelin’s values have been found to be increased in patients with chronic liver disease [5]. Salusins, which were discovered in 2002, are soluble peptide hormones formed from prosalusin precursors [6]. They are divided into two known peptides, composed of 28 and 20 amino acids, called salusin-alpha and salusin-beta. Their biological function is based on their mild hypotensive, anti-sclerotic and chronotropic negative effect [7]. Previous studies of apelin and salusin performed in pregnant women with hypertension have given conflicting results. In some of the prelabeling work, the concentration of apelin is higher than in the control groups. In other studies, the concentration of this substance in pregnancies complicated with hypertension was lower than in healthy pregnancies. There has been no study on the above-mentioned substances in other forms of hypertension, or in obese pregnant women. 

In many scientific reports, the usefulness of assessing the occurrence of cffDNA in the mother’s circulation when screening for fetal genetic defects has been confirmed [8]. Currently, attempts are being made to use these techniques to assess the risk of other pregnancy pathologies, especially in obese women. One of the elements associated with placental disorders in preeclampsia is the increase in the plasma concentration of free fetal DNA in the maternal circulation [9,10]. The presence of fetal DNA in the maternal circulation was noted by the detection of specific nucleotide sequences on the Y-chromosome (SRY genotyping, sex-determining Y region) [11,12]. Unfortunately, SRY genotyping has the disadvantage of only being able to evaluate fetal DNA in male fetuses [13]. However, there are reports that detect SRY in pregnancies with female fetuses or placental mosaicism [14,15]. This problem is solved by the detection of cffDNA by fetal DNA methylation in the area of the maspin gene in fetal tissues, or by the detection of the amelogenin gene in cffDNA [16,17]. The amelogenin gene is located on X- and Y-chromosomes at Xp22.1–Xp22.3 and Yp 11.2. The amelogenin gene (AMELX and AMELY) allows us to determine the sex of unknown human samples, although AMELY varies among individuals and cases [18]. 

Maternal obesity is a major health concern. According to the WHO in Europe, 54.3% of women over the age of 18 are overweight, and 24.5% of them are obese [19]. Multiple studies have found an association between increasing maternal weight and decreases in cell-free fetal DNA in Non-Invasive Prenatal Testing (NIPT) [20].

This study aimed to determine if obesity could influence the percentage of cffDNA in preeclamptic women, and if this effect could be mediated by the disturbance of hypotensive mechanisms.

## 2. Materials and Methods

The pregnant women hospitalized between 2008 and 2015 in the Department of Obstetrics and Pathology of Pregnancy in Lublin, Poland were divided into groups: a control group (C) with 67 pregnant women, a gestational hypertension group (GH) with 35, and group with preeclampsia (PE) with 66 patients. The samples were taken for investigation between the 25th and 41st weeks’ gestation.

The women with preeclampsia were further divided into two subgroups: obese (O) (pre-gravid BMI > 30) and lean (pre-gravid BMI < 25). The first subgroup contained 23 pregnant women and the second included 43. All pregnant women with diagnosed hypertension received methyldopa at therapeutic doses to control their blood pressure.

The general criteria for selecting the patients undergoing the examination were as follows:

1. Normal blood pressure before pregnancy.
2. No general diseases (patients with diabetes, thyroid, kidney diseases or connective tissue disorders were excluded).
3. A physiological single pregnancy (pregnant women with threatened premature delivery, gestational diabetes or thrombocytopenia were excluded).
4. No fetal structural or chromosomal abnormalities detected in prenatal tests and after pregnancy.
5. The occurrence of hypertension ≥ 140/90 mmHg after the 20th week of pregnancy.
6. A proteinuria of 1+ or higher in a single urine sample, or ≥300 mg in a 24-h urine collection.
7. No history of hypertensive disorders during previous pregnancies.
8. Body mass index calculation was defined as the body mass divided by the square of the body height, in units of kg/m^2^.

The criteria for including patients in the study PE group were determined as follows:

- Hypertension was first diagnosed after the 20th week of pregnancy.
- Proteinuria was found.
- The presence of other systemic or fetal abnormalities was excluded.
- Pre-gravid BMI < 25.

The criteria for preeclampsia with the obesity group included all of the above as well as a pre-gravid body mass index over 30.

The criteria for including lean patients (BMI < 25) in the group with gestational hypertension were as follows:

- Hypertension was first diagnosed after the 20th week of pregnancy.
- The presence of other systemic or fetal abnormalities was excluded.
- Absence of significant proteinuria.

The criteria for the control group:

1. Normal blood pressure before pregnancy.

9. No systemic diseases.
10. The current correct course of a single pregnancy.
11. No fetal structural or chromosomal abnormalities detected in prenatal tests and after pregnancy.
12. No history of hypertensive disorders during previous pregnancies.
13. Pre-gravid BMI < 25.

In 33 patients, the mild form of preeclampsia was present. The same number of pregnant women developed a severe form of this disease. Division into two groups was carried out based on the occurrence of one or more of the following symptoms (Table 1):

In seven patients, the first symptoms of hypertension and proteinuria were observed before 34 weeks of gestation. The rest of the pregnant women had late preeclampsia (59 patients).

The research material was the blood serum and oral mucosa swabs obtained from 168 Caucasian patients hospitalized in the Department of Obstetrics and Pregnancy Pathology at the Medical University of Lublin. All the patients were analyzed for apelin, salusin-alpha, prosalusin and cell-free fetal DNA.

The research material was pregnant plasma collected from the patients who qualified for the study. In order to verify the genetic material, a swab was taken from the mucosa of the inner side of the cheek in the mother and from the newborn after delivery. The test material was plasma, obtained from the blood of the patients and collected on citrate anticoagulant (0.109 M). In order to separate the plasma from the morphotic elements, the whole blood was centrifuged twice using a rotor, for 15 min at the angular velocity of 2500× *g*. In order to extract the microparticles, the centrifuged samples were centrifuged again for 90 min at 16,000× *g*. The microparticles were centrifuged in the lower fraction of the sample after centrifugation.

The determination of the human prosalusin level was performed using the ELISA Human ELISA Kit (catalog number: E1892h) from Wuhan EIAab Science Co., LTD (Wuhan, China). The measuring scale ranged from 0.31 to 20.0 ng/mL. The concentration of Apelin-36 and Salusin-α in the blood serum was determined using test kits from Phoenix Europe GmbH (Mannheim, Germany), namely the Apelin-36 (Human) EIA Kit, catalog number: EK-057-15. The analytical sensitivity of the test was 0.1 ng/mL, and the measurement scale ranged from 0 to 100 ng/mL. Salusin-α (Human) EIA Kit, Catalog No.: EK-010-67 The analytical sensitivity of the test was 0.25 ng/mL and the measurement scale was within the range of from 0 to 100 ng/mL.

The tests were carried out according to the procedure attached by the manufacturer of each set. The results were read in a Microplate Reader model 680 plate reader (BioRad, Hemel Hempstead UK). 

### 2.1. DNA Preparation

Samples were stored at –80 °C and frozen in liquid nitrogen for isolation. Total cellular DNA was isolated and purified from about 200 mL of blood using commercially available DNA isolation kits DNeasy/RNeasy Mini Kitnr (QIAGEN, Germantown, NY, USA) according to the manufacturer’s instructions. The DNA quality was estimated by agarose gel electrophoresis.

### 2.2. CffDNA Genotyping

Based on the polymorphism analysis of the autosomal short tandem repeat (STR), microsatellite loci and genes were located in the Y-chromosome. Such repetitions occur in a population of a variable number (from 5 to 100). The AmpFLISTER NGM PCR Amplification Kit (Thermo Fisher Scientific, Waltham, MA, USA) was used for amplification of the DNA, allowing for analysis of the following microsatellite repeats: D2S1338, D3S1358, D8S1179, D16S539, D18S51, D19S433, D21S11 (located on different chromosomes in the human genome) and, in the FGA gene (fibrinogen), vWA (von Willbrand factor) and TH01 (tyrosine heptaxylase). Genotyping was performed using an ABI 310 analyzer (Applied Biosystems, Waltham, MA, USA).

### 2.3. Genotyping STR

In order to determine the STR genotype for the 15 microsatellite loci and amelogenin of the sex marker when establishing the biological material of the offspring of the mothers, the AmpFlSTR NGM PCR Kit (Thermo Fisher Scientific) was used. The PCR reaction was carried out in a final volume of 5 μL containing 1× AmpFlSTR NGM Master Mix, 1× AmpFlSTR NGM DNA primers and, quantitatively, ranging from 0.5 to 2 ng. In the case of a low concentration of the entry DNA matrix, the volume of the PCR reaction was increased to 10 μL to ensure an adequate amount of template in the reaction mixture. The conditions of the thermal cycles were in accordance with the manufacturer’s recommendations. An analysis of the size of the PCR products was carried out in an ABI 3130xl automatic sequencing apparatus; (POP-7 gel filter set G5 length 26 cm, software GeneMapper version 5.0). The size of the PCR products was determined in relation to the internal standard size of GS600LIZ (Thermo Fisher Scientific, Waltham, MA, USA).

Identification of the coding sequence of the SRY9MBI Fermentas0 gene: the SRY gene is located on the Y-chromosome and encodes a transcription factor that initiates the activation of genes responsible for male gender differentiation. The DNA, isolated from the peripheral blood, was a matrix to identify the coding sequence of a gene in a PCR reaction. SRY F: 5′GAATATTCCCCGCTCTCCGGAG-3′ and SRY R: 5′-ACCTGTTGTCCAGTTGCACT-3′ were used to amplify a 418 bp fragment. The PCR mixture contained 0.2 ug of genomic DNA, 2.5 U of Taq polymerase, 0.5 umol/L of each primer, 100 mm/L dNTPs, 3.0 Mm MgCl2 and 1× PCR buffer in a final volume of 50 µL. The PCR condition was 2 min at 94 °C for pre-heating, 35 cycles of 94 °C for 30 s, 570 °C for 30 s and 72 °C for 1 min and 72 °C for 10 min, as described (Erdal, M.E.; Barlas, I.O. Detection of the SRY Gene in a 46, XY Phenotypic Female by the PCR-SSCP Method. *Turk. J. Med. Sci*. **2000**, *30*, 501–503). The products of amplifications were separated in 1.55 agarose gel and by sequencing.

The conduct of the clinical trials was accepted by the Bioethics Committee of the Medical University of Lublin (no. KE-0254/234/2015 date 25 June 2015, and KE-0254/165/2012 date 28 June 2012) Each patient received an informed consent form and a standardized questionnaire.

### 2.4. Statistical Analysis

A statistical analysis of clinical data was carried out using the arithmetic mean standard deviation (SD) medians (ME) using the Shapiro test and the Kruskal–Wallis H test. A comparison of the differences between the control group and the study group, and between the subgroups of patients, was carried out with the *t*-student test. For the variables tested, which did not show normal distribution, non-parametric tests were used for further analyses. The concentrations of apelin, salusin-alpha, prosalusin were compared using the U-Mann–Whitney test and the Pearson Chi2 test. The relationship between individual substances and clinical data was found using the Spearman correlation test. Statistical significance was set at *p* ≤ 0.05. Results which were statistically insignificant were defined by the abbreviation “ns”. Statistical calculations were based on Statistica 10 (StatSoft, Tulsa, OK, USA).

## 3. Results

The study involved 168 pregnant women, who were divided into three groups:

- Control group—67 patients (39.88%);
- Patients with PE—66 patients (39.29%): including obese patients (PE + O) (pre-gravid BMI > 30)—23 patients (34.85%)—and lean with PE—43 patients (65.15%);
- Patients with GH—35 patients (20.83%).

A general statistical analysis was performed of baseline characteristics (Table 2). These characteristics show:

Maternal age did not differ between groups (Kruskal–Wallis H test = 2.24, *p* = 0.33); vaginal labor was significantly more frequent in the control group, while there were more caesarean sections in the PE, obesity, and GH group (Pearson Chi2 = 30.98, df = 2, *p* = 0.000001); the gestity in the individual groups of patients did not differ significantly (Kruskal–Wallis H = 0.02, *p* = 0.99); the parity in the individual groups of patients did not differ significantly (Kruskal–Wallis H = 1.54, *p* = 0.46); term at delivery was significantly different between groups. (Kruskal–Wallis H test = 37.10, *p* = 0.00001); the newborn weight was statistically significantly different between groups (Kruskal–Wallis H test = 11.86, *p* = 0.003). Statistically significant differences occurred between the birth weight of the child of the control patient and the PE and obesity groups.

The study plan for our research was to assess the influence of maternal obesity on cffDNA and the effectiveness of hypotension mechanisms between the control group and pregnancies with hypertensive disorders.

**Figure 1:**
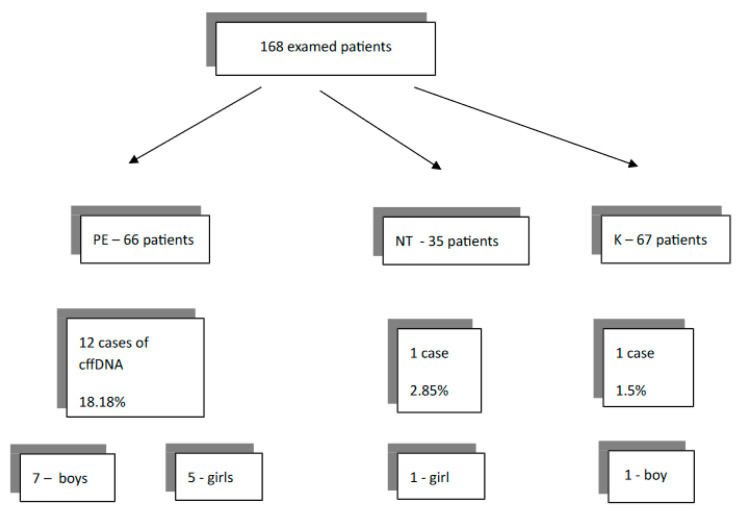
Results of assessing the occurrence of cffDNA in pregnant plasma.

An assessment of the number of the cases with cffDNA in maternal circulation was made. A summary is presented graphically in Figure 1.

The results were as follows. In 1.50% of patients in the control group, the cffDNA was detected. In the gestational hypertension group, this ratio was 2.85%. A significant elevation of cffDNA to 18.18% was observed in PE patients. Obesity had a statistically significant influence on cffDNA in the subgroup of pregnant women with PE. The fetal DNA was altered in 7 out of 12 cases (58%).

There were no statistically significant correlations between obesity and early, late, or severe preeclampsia and the occurrence of cffDNA (Chi2 Yates test = 3.43, df = 2, *p* = 0.18).

In the next step of the analysis, the correlation between the levels of salusin-alpha, prosalusin, apelin, and presence of fetal-free DNA in the maternal circulation were evaluated in all groups. Variables that had a statistically significant effect on the occurrence of cffDNA are shown in Table 3.

**Figure 2:**
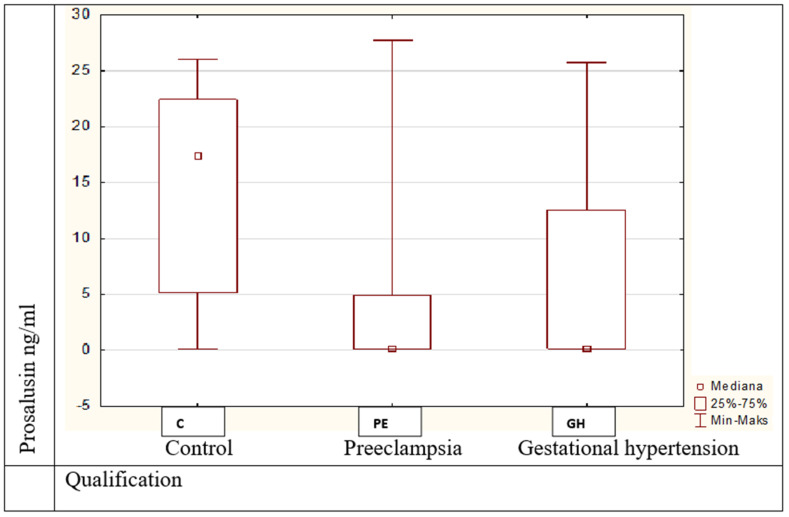
Prosalusin values between pregnant women in studied groups, *p* = 0.00001.

**Figure 3:**
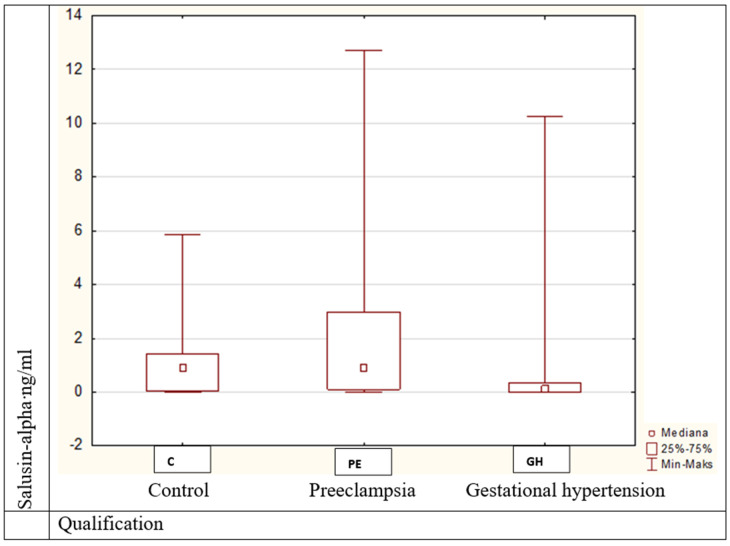
Salusin-alpha values in pregnant women between the examined groups, *p* = 0.003.

Finally, the tested substances were assessed, comparing the study groups. The following relationships were found:

(1). The values of prosalusin were statistically significantly different (Kruskal–Wallis H test = 43.37, *p* = 0.00001) between the control group and obesity with preeclampsia and between the control and the group with gestational hypertension (Figure 2);

(2). Salusin-alpha values were statistically significantly different (Kruskal–Wallis H = 11.43, *p* = 0.003) between patients with obesity and preeclampsia and those with gestational hypertension (Figure 3);

(3). Apelin’s values differed statistically significantly (Kruskal–Wallis H = 9.85, *p* = 0.007).

**Figure 4:**
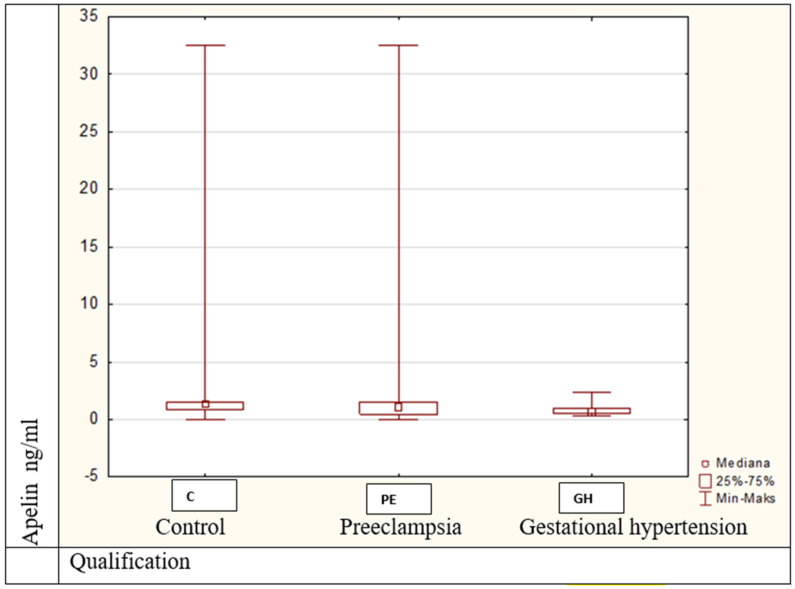
Apelin values between pregnant women in all groups, *p* = 0.007.

Statistically significant differences were noted between controls and preeclamptic women (Figure 4).

## 4. Discussion

Obesity is a major health concern, especially in women with hypertensive disorders [21]; it is of particular concern in pregnancy due to heightened risks for mother and/or baby. Preeclampsia is characterized by a complex pathophysiology, which involves many systems of proteins, cytokines, hormones and metabolic processes. Gestational hypertension is a different condition than preeclampsia; thus, an astute and circumspect diagnostic approach is required.

The prevalence of obesity is increasing worldwide; however, Polish society has one of the lowest percentages in Europe (7.1%) [22]. Therefore, our research was the first to be carried out on such a large and uniform group of patients. The study involved 168 patients, who were divided into three major groups and two subgroups: obese (O) (pre-gravid BMI > 30) and lean patients (pre-gravid BMI < 25), and in which the concentrations of apelin, salusin-alpha, prosalusin, and cell-free fetal DNA in the mother’s plasma were analyzed. The impact of obesity on the mechanisms of hypotension, and the incidence of fetal DNA in maternal plasma, are also unique research topics.

This study has shown correlations between obesity and the mechanisms responsible for blood pressure control. The studied proteins were chosen for analysis because of their properties of vasodilation and blood pressure regulation. The salusins are bioactive peptides, with hemodynamic effects. Their role in hypertension, atherogenesis and cardiovascular diseases is related to the regulation of blood pressure. Apelin causes systemic vasodilatation and increased cardiac contractility in humans. In our study, it was confirmed that an increased BMI in PE increases the concentration of apelin.

Studies have confirmed the involvement of apelin in the process of regulating blood pressure in animals as well as in humans [3]. Apelin modulates the contractility of myocardial and blood vessel tissue [23]. The role of apelin in physiological pregnancy is limited to the regulation of implantation and placement [3]. In the first trimester, apelin is found in the cytoplasm of cytotrophoblast cells, and forms the inner proliferative placental villi and, in small concentrations, in syncytiotrophoblast cells. In the third trimester of pregnancy, the expression of apelin decreases and this protein occurs almost exclusively in the cytotrophoblast. Preeclampsia modulates the apelinergic system, causing an increase in apelin in the stroma and on the trophoblastic surface. The expression of apelin in endothelial cells causes paracrine signals to smooth the muscle cells of the blood vessels, with potential vasoconstrictive effects.

Our study found higher concentrations of apelin in patients with preeclampsia. Similar results to ours were observed by other researchers [24,25,26]. In another study, these results were questioned, and a reduction in apelin values was demonstrated in pregnant women with preeclampsia [6,27]. It seems that the reduced apelin concentration in the placenta may affect the migration of trophoblast cells along the spiral arteries and interfere with the proper vascularization, not only in preeclampsia but also in FGR [28,29]. Elevated maternal plasma apelin values may come from a source other than the placenta, such as fat tissue, or be a response to elevated vascular resistance or hypertension induced by factors other than apelin [26]. The role of the endogenous apelin release system is still not fully understood. However, because it takes part in angiogenesis, APJ is involved in the pathogenesis of PE [30]. Apelin is currently used to treat preeclampsia on animal models [31,32]. To date, there have been no studies on the effect of apelin on the occurrence of fetal DNA leakage into the mother’s circulation. Observations made by our team confirm that fetal DNA is more common in pregnant women with preeclampsia who have a higher concentration of apelin. It seems that less efficient vasodilating mechanisms co-exist, causing greater damage to the endothelium and leading to the detection of fetal DNA. Our studies also pointed to higher levels of apelin in the preeclampsia subgroup with obesity. These findings are the first to be reported and need further investigation. However, recently, a study by Motawi et al. confirmed a significant correlation between apelin and obese type 2 diabetic patients with coronary artery stenosis [33].

Salusins and their precursor, prosalusin, are multifunctional bioactive peptides, synthesized in many human tissues, lowering hypertension without affecting the synthesis of nitric oxide. The evaluation of the concentration of the above substances in our study aimed to determine their impact on the course of preeclampsia, which is characterized by an increased contraction of blood vessels, hypoperfusion and tissue necrosis. In several publications on the occurrence of salusin, slightly higher values were found in pregnant women with preeclampsia than in physiological pregnancies [24]. In our results, this relationship was not confirmed, but there were statistically significant differences in salusin-alpha concentrations between lean or obese patients with preeclampsia and gestational hypertension. To date, salusin concentrations have not been analyzed in pregnant patients with hypertension without proteinuria nor obesity. An analysis of plasma salusin-alpha concentrations in patients with chronic hypertension and atherosclerosis of the cervical vessels revealed a decrease in its concentration [7]. The authors argue that plasma salusin-alpha concentrations may not be directly controlled by the physiological mechanisms that lower blood pressure. In addition, salusins play a smaller role in maintaining normal blood pressure values, and their mechanism of action is not clearly understood. Presumably, the higher values of salusin-alpha recorded in the pregnancies we examined may be a form of the body’s adaptation to vasoconstriction, which speaks to another pathophysiology of the development of blood pressure disorders, with or without proteinuria. Our analysis attempted, for the first time, to determine the relationship between the occurrence of cffDNA and the salusin-alpha concentration, and no statistically significant differences were found. The probable reason for this is another vasodilator mechanism similar to apelin, for which the results were positive. Due to the ambiguous results of the research, the role of salusin in the pathophysiology of preeclampsia and obesity merits further study.

The precursor to salusin in the human body is prosalusin, and our study is the first one to determine the concentration of prosalusin in pregnancies with abnormal blood pressure during pregnancy. It was observed that its values were, statistically, the lowest in the group with preeclampsia as compared to the control group and that with gestational hypertension. In addition, there was a correlation between the occurrence of fetal DNA and the low concentration of prosalusin in preeclamptic pregnancies. This is confirmed by the failure of mechanisms that lower blood pressure with a co-existing loss of protein. However, these results are the opposite of those found for salusin-alpha. Further studies should be carried out to determine how preeclampsia affects the biosynthesis of this protein.

Cell-free fetal DNA may provide information about the placenta, and potentially be used to predict clinical problems. Studies on the first trimester of pregnancy and cffDNA predictive properties provide conflicting results. According to some researchers, the assessment of fetal DNA between 11–13 and 20–24 weeks of pregnancy is not a good predictor of preeclampsia [10,34,35]. However, other studies, although on smaller groups of patients, provide opposing results [36]. In subsequent trimesters of pregnancy, especially in cases of severe and early preeclampsia and FGR, the results almost unanimously confirm the increased amount of fetal DNA in mothers with a state of impaired placement [9,37]. The occurrence of cffDNA was also confirmed 3 weeks before the onset of symptoms of preeclampsia [38]. In our study material, a more frequent occurrence of cffDNA in pregnancy with preeclampsia was confirmed, in comparison to the control group and pregnancies with hypertension (18.18% vs. 1.50% vs. 2.85%). Similar to other researchers, we assume that the higher maternal serum of cfDNA may reflect an increased systemic activation of maternal inflammation in preeclampsia [39].

In a study established on a mouse model of maternal-diet-induced obesity, the placentas from obese dams released significantly less cfDNA compared to placentas from lean dams at time 0 in culture [40]. According to the researchers, this reflected the in vivo physiology of placental cfDNA release just prior to parturition. However, at later timepoints, there were no significant differences between obese and lean placentas at 1 h and 6 h. This might be more reflective of cellular changes/apoptosis of fetal tissues. The amount of cfDNA released by the placenta was not influenced by fetal sex in this study. These animal data suggested a refined hypothesis for the lower fetal fraction of cfDNA observed in maternal obesity. In opposition to this study, in our sub-group of preeclampsia and obesity, cell-free fetal DNA was observed in 58% of patients, which is statistically significant. In other research, it became understood that increased amounts of maternal cell-free DNA in the circulation of obese pregnant women may result from the adipocyte necrosis or apoptosis of placental cells [41].

In the latest study, the association between cell-free DNA and gestational diabetes (GDM) in a cohort of women presenting for screening for fetal aneuploidy was determined. Although fetal fraction was lower among women diagnosed with GDM, this relationship was not statistically significant in obese patients. The researchers associate obesity with a lower cell-free fetal fraction, as a result of the dilution effect on the placental cfDNA due to an increased amount of maternal cfDNA. Maternal cfDNA levels might be increased in obese women as a result of greater necrosis and apoptosis of adipose tissue and an increased maternal plasma volume. Obesity is a known risk factor for GDM; therefore, for the authors, there was no association between fetal fraction of cfDNA and the development of GDM [42].

Our research is the first to estimate the increase in purely fetal cffDNA due to obesity with preeclampsia patients.

The limitations of many research tests are the possibility of determining fetal DNA only in pregnancies with a male fetus. In our study, the determination of amelogenin allowed us to avoid omitting female fetuses. It has been suggested that the test determining the sex with the amelogenin gene is usually sufficient, while other markers of the Y-chromosome (SRY, STR, 50f2) can be used to identify gender in more ambiguous cases [43]. The misidentification rate using amelogenin appears to be low. In one study in Spain, the amelogenin sex determination test using AMELX (977 bps) and AMELY (790 bps) bands was performed for 1224 individuals of known gender, with a 99.84% (1222/1224) accuracy rate [17]. Our study is the first to detect male and female cffDNA in serum in patients with preeclampsia and gestational hypertension with normal and increased BMI.

Another limitation of our research is the performance of analyzes in obese patients *per se*. Obesity is a multifactorial disease, associated with complex metabolic and physiological changes. Adipose tissue is not only fat storage but rather a hormonally active tissue producing cytokines, e.g., adipokines. Our study did not explore which substance or mechanism is responsible for increasing the risk of preeclampsia, placental damage nor blood pressure regulation. The BMI index itself is also is an imperfect indicator for adiposity. The measurement of body composition, including body fat percentage, might identify the obese woman at risk of preeclampsia more accurately.

In sum, since there is no effective treatment for preeclampsia other than delivery to improve the prenatal care standard, low-dose aspirin prophylaxis is the most useful preventive pharmacologic intervention, especially in pregnant women with obesity.

## 5. Conclusions

Obesity in preeclampsia is an independent risk factor for placental damage and disturbances in maintaining normal blood pressure. This study found the presence of cell-free fetal DNA in the serum of pregnant women with obesity and preeclampsia to be significantly more common and associated with inefficient hypotensive mechanisms. Understanding the pathomechanism of hypertension in pregnancy is crucial in the development of new therapeutic strategies against preeclampsia.

## Figures and Tables

**Table 1:** Division of symptoms of mild and severe preeclampsia.

	Mild Preeclampsia	Severe Preeclampsia
Systolic pressure	140 mmHg	160 mmHg
Diastolic pressure	90 mmHg	110 mmHg
Proteinuria	≥300 mg/24 h or ≤+2	≥5 g/24 h or >+2
General symptoms (pulmonary oedema, oliguria, headache, etc.): rise in creatinine (90 micromol/liter or more, 1.1 mg/100 mL or more) or rise in alanine transaminase (over 70 IU/liter, or twice upper limit of normal range) or fall in platelet count (under 100,000/microliter)	None	Present

**Table 2:** Baseline characteristics.

	Maternal Age (Years), Mean ± SD	Delivery in Weeks, Mean ± SD	Vaginal Delivery/C-Section	Fetal Weight (Grams), Mean ± SD
C	18–42, 29.09 ± 5.08	37–42, 39.32 ± 2.37	62.69%/32.84%	2230–4330, 3422.62 ± 406.24
GH	21–38, 30.51 ± 4.09	29–42, 37 ± 3.32	25.71%/74.29%	1140–5440, 3317.14 ± 791.13
PE	19–42, 30.59 ± 4.94	25–41, 36.97 ± 3.15	19.40%/80.60%	780–4480, 2897.16 ± 816.56
PE + O	19–40, 29.7 ± 5.11	31–41, 37.95 ± 2.44	21,73/78.26%	2630–4500, 3496.13 ± 506.60

C—control group, GH—gestational hypertension, PE—preeclampsia group, PE + O—preeclampsia and obesity subgroup.

**Table 3:** The values of studied substances and their statistical significance.

Name and Presents of Substances	Range (ng/mL)	Median	Mann–Whitney Test Z Score	*p* Value
Prosalusin in sera with cffDNA	0.11–8.51	0.14	−2.26	0.008
Prosalusin without cffDNA	0.11–27.75	0.28		
Salusin in sera with cffDNA	0.47–3.98	1.65	1.11	0.27
Salusin without cffDNA	0.012–2.98	0.64		
Apelin in sera with cffDNA	0.80–2.60	1.53	2.76	0.006
Apelin without cffDNA	0.009–32.54	0.84		

## Abbreviations

BMI	body mass index
PE	preeclampsia
PE + O	preeclampsia and obesity
GH	gestational hypertension,
FGR	fetal growth restriction
GDM	gestational diabetes
cffDNA	cell-free fetal DNA
cfDNA	cell-free DNA (mothers)
STR	short tandem repeat
SRY	sex-determining *region* Y
